# Biodegradable suture development-based albumin composites for tissue engineering applications

**DOI:** 10.1038/s41598-024-58194-5

**Published:** 2024-04-04

**Authors:** Mohamed A. Naser, Ahmed M. Sayed, Wael Abdelmoez, Mohamed Tarek El-Wakad, Mohamed S. Abdo

**Affiliations:** 1 Faculty of Engineering, Biomedical Engineering Department, Minia University, Minia, Egypt; 2 Faculty of Engineering, Biomedical Engineering Department, Helwan University, Helwan, Egypt; 3 Faculty of Engineering, Chemical Engineering Department, Minia University, Minia, Egypt; 4 Faculty of Engineering and Technology, Future University Egypt, Fifth Settlement, Cairo Egypt; 5 EECS Department, MSOE University, Milwaukee, United States

**Keywords:** Biodegradable materials, Surgical sutures, Serum albumin, Extrusion process, Tissue engineering applications, Biomaterials, Nanobiotechnology, Tissue engineering

## Abstract

Recent advancements in the field of biomedical engineering have underscored the pivotal role of biodegradable materials in addressing the challenges associated with tissue regeneration therapies. The spectrum of biodegradable materials presently encompasses ceramics, polymers, metals, and composites, each offering distinct advantages for the replacement or repair of compromised human tissues. Despite their utility, these biomaterials are not devoid of limitations, with issues such as suboptimal tissue integration, potential cytotoxicity, and mechanical mismatch (stress shielding) emerging as significant concerns. To mitigate these drawbacks, our research collective has embarked on the development of protein-based composite materials, showcasing enhanced biodegradability and biocompatibility. This study is dedicated to the elaboration and characterization of an innovative suture fabricated from human serum albumin through an extrusion methodology. Employing a suite of analytical techniques—namely tensile testing, scanning electron microscopy (SEM), and thermal gravimetric analysis (TGA)—we endeavored to elucidate the physicochemical attributes of the engineered suture. Additionally, the investigation extends to assessing the influence of integrating biodegradable organic modifiers on the suture's mechanical performance. Preliminary tensile testing has delineated the mechanical profile of the Filament Suture (FS), delineating tensile strengths spanning 1.3 to 9.616 MPa and elongation at break percentages ranging from 11.5 to 146.64%. These findings illuminate the mechanical versatility of the suture, hinting at its applicability across a broad spectrum of medical interventions. Subsequent analyses via SEM and TGA are anticipated to further delineate the suture’s morphological features and thermal resilience, thereby enriching our comprehension of its overall performance characteristics. Moreover, the investigation delves into the ramifications of incorporating biodegradable organic constituents on the suture's mechanical integrity. Collectively, the study not only sheds light on the mechanical and thermal dynamics of a novel suture material derived from human serum albumin but also explores the prospective enhancements afforded by the amalgamation of biodegradable organic compounds, thereby broadening the horizon for future biomedical applications.

## Introduction

Biomaterial polymer research has been prioritized to accelerate tissue regeneration, as biopolymers are biocompatible and can combine with the human tissue surrounding the biomaterial. Polymeric materials show unique features, including good mechanical properties and easy to synthesis, which makes them suitable for organ scaffold development and production. However, only few polymers have shown proper biocompatibility and consistency with other matrix proteins and growth factors, as in vitro and in vivo* studies* have shown^1^. Many protein-derived biomaterials, such as collagen, elastin, and casein, have been extensively employed in various applications to enhance cell growth and thus, can be used to develop new biomaterials for biomedical applications^2,3^ .

Surgical sutures can be natural or synthetic. Surgical sutures have grown rapidly in the last two decades, becoming the largest group of biomaterials with a market worth of more than $1.3 billion yearly^4,5^. Synthetic sutures are produced from a range of textiles developed specifically for surgical use, such as nylon or polyester. In 2006, Li and Yuan ^6^ reported a review on the scientific advances in synthetic absorbable polymeric sutures. Hon et al. ^7^ reported a comparative study of several closure materials used in vascular devices in 2009. They provided a review of sutures used in vascular devices, including how they work, how effective they are at attaining hemostasis, any hazards associated with their usage, and which sutures should be utilized for specific purposes. Gassner ^8^ conducted a review of wound closure materials in 2002 that summarized the side effects of sutures. Singhal et al. reported a review study that extensively focused on biodegradability and sutures trends^9^. They highlighted the key characteristics of absorbable sutures made from poly glycolic acid (PGA) and its copolymers, as well as the upcoming polydioxanone (PDO; some authors used PDS to refer to polydioxanone sutures) and poly trimethylene carbonate (PTMC)-based absorbable sutures. Polyglycolic acid or other glycolide polymers were used to make resorbable synthetic sutures. Many surgical sutures are made of the water-resistant material Gore-Tex, whereas other sutures are formed of thin metal wires^10,11^.

Surgical sutures can be divided into two types: absorbable and non-absorbable^5,6^. Non-absorbable sutures are used to provide long-term tissue support, remaining walled-off by the body’s inflammatory processes (until removed manually). Non-absorbable sutures often cause less scarring because they provoke less immune response, and thus are used where cosmetic outcome is essential. They may be removed after a specific time or left permanently^13^.

Absorbable suture materials include the original catgut as well as the newer synthetics polyglycolic acid, polylactic acid, polydioxanone, and caprolactone. The utilization of biodegradable sutures provides several benefits in tissue engineering. The need for a second surgery to remove them is eliminated, thus reducing the risk of infection associated with the presence of foreign material in the body. Due to the fact that biodegradable sutures decompose over time, they reduce the risk of tissue damage or trauma during suture removal, which is particularly advantageous in delicate tissues or in areas where repeated surgeries may be required. Biodegradable sutures serve as a scaffold for tissue regeneration and can be tailored to degrade at a rate that aligns with the healing process of the tissue, thereby facilitating natural healing and mitigating inflammation. These sutures are frequently composed of materials that are well tolerated by the body, such as polymers like polylactic acid (PLA) or polyglycolic acid (PGA), which possess biocompatibility and gradually degrade into non-toxic byproducts. Customization of biodegradable sutures for specific applications can be achieved by adjusting the composition and structure of the material, thereby meeting the requirements of the tissue engineering application. In comparison to permanent sutures, biodegradable sutures are able to minimize the foreign body response, thus decreasing the risk of chronic inflammation and tissue rejection. Due to the natural degradation of biodegradable sutures over time, the need for suture removal is eliminated, resulting in reduced patient discomfort, and simplified postoperative care. Although the initial cost of biodegradable sutures may be higher than that of traditional sutures, the avoidance of secondary removal procedures and potential complications associated with permanent sutures can result in long-term cost savings ^14–16^. In general, the utilization of biodegradable sutures in tissue engineering presents a variety of advantages, such as enhanced biocompatibility, reduced infection risk, improved healing, and simplified postoperative care, which makes them an appealing choice for various surgical and tissue engineering applications. In characterizing the mechanical properties of sutures, tensile strength and elongation percentage are frequently measured. Mechanical strength is the most commonly referenced physical feature of sutures^5,17,18^.

While there may be limited research on the development of sutures using albumin-based composites, there are studies that investigate the use of these materials in tissue engineering and suturing applications. Pourali et al.^19^ conducted a study that specifically examines the development of albumin-based nanoparticles for suture applications. The researchers synthesized biodegradable nanoparticles using albumin as the main component and evaluated their mechanical properties, biocompatibility, and degradation kinetics. The results showed promising potential for these albumin-based composites to be used as suture materials in tissue engineering due to their biocompatibility and tunable degradation properties.

The review by Müller et al.^20^ reports the utilization of various biodegradable sutures in orthopedic surgery, including their benefits and limitations, although it does not specifically focus on albumin-based composites. The review emphasizes the significance of employing materials that degrade over time, thereby minimizing the requirement for suture removal and reducing tissue trauma. Within this context, albumin-based composites could be investigated for their potential advantages in orthopedic suture applications. Zhou et al.^21^ discovered that coating absorbable sutures with albumin enhanced cell attachment and proliferation, rendering them suitable for delivering cell therapy in soft tissues. Another study was conducted to develop silk sutures that were coated with silk fibroin films containing berberine. These sutures exhibited sustainable antibacterial properties and demonstrated good mechanical and biocompatible properties ^22^. Furthermore, a novel surgical suture was documented that integrated natural antibacterial berberine into shape memory polyurethane fibers, resulting in antibacterial activity and shape memory effect^23^. These findings indicate that the use of albumin-based sutures has the potential to enhance the healing process of wounds, prevent infections, and improve the outcomes of surgical procedures.

The key objective of this study is to prepare Filament Suture (FS) from protein-based serum albumin^24–26^ using the extrusion process. We developed a new method that can improve the mechanical, biological, and chemical proprieties of absorbable suture materials. The proposed FS can be used as filaments for 3-D printers for many applications, such as plates and nails. The FS was characterized for its physio-chemical properties using scanning electron microscopy (SEM) and thermogravimetric analysis (TGA). The studied mechanical parameters were tensile strength and elongation at break. We show that using protein of human serum albumin (PHSA) can be exploited in the development of new biodegradable sutures that can be used in many medical applications.

## Materials and methods

### Base material

Among various types of polymers, natural polymers are considered the most fitting due to their remarkable biocompatibility, appropriate interactions, and propensity for biodegradability. In order to attain this objective, polymer materials must possess characteristics such as transparency, permeability to water and oxygen, while simultaneously being impervious to bacteria. Additionally, they must create a secure and moist environment^17,27,28^. Human serum albumin (HSA) is a protein found in serum albumin. In laboratory research, it is frequently used as a reference for protein concentration. HSA has many biochemical uses, notably in immunoblots, immunohistochemistry, and enzyme-linked immunosorbent assays. HSA is an excellent replacement for human serum albumin (HSA) in laboratory experiments because of its structural similarity. The polypeptide chain of the protein, which contains 585 amino acid residues, is folded into three -helical domains to retain the protein's heart-shaped structure. A lot of ligands and metal ions can be reversibly bound to albumin^29,30^.

This study presents the synthesis of a new biodegradable suture based on human serum albumin (HSA) produced using sub-critical water technology. Previously, Abdelmoez et al.^23^ demonstrated the theory of protein synthesis from HSA using sub-critical water technology. The pure HSA (first grade) and other chemicals used in the initial preparation trials were provided by Wako Pure Chemical Industries, Ltd. (Osaka, Japan)^23,24^. When heated, HSA forms soluble aggregates of polymerized molecules via desulphation process and through creation of noncovalent bonds during the early stages of heat-induced gelation of the protein.

### Additive materials

Highly efficient biocompatible polymers were chosen as additive materials for suture synthesis, based on prior studies in the literature ^6,10,12,13,31^. We used the following four additives:

1. Gelatin powder: a powder used for many biomedical applications at 1–5 μg/cm2 or 0.5–50 μg/mL concentration. It has a viscosity of 30–48 mpa•s, pH: 5.5–7, Jelly strength :120—260 bloom, Molar Mass: 50,000–60,000 (Qualikems, India, Empirical formula: C102H151N31O39).
  Due to its reversible triple helix shape at low temperatures, gelatin exhibits significant potential for use in the creation of thermo-responsive biomaterials. Since gelatin lacks stable molecular entanglement as net-points in the polymer structure. As a consequence, the mixture of gelatin and materials containing functional group may be promising for biosynthesis of hydrogels that meet the requirements of advanced materials for wound closure and healing^32^.
2. Polyvinyl alcohol (PVA): it is a biocompatible, hydrophilic, and biodegradable polymer that can be used in drug delivery and tissue engineering applications. This alcohol has an average molecular weight of: 30,000–70,000, viscosity: 50.0 mpa•s, degree of hydrolysis 80% mol/mol, PH value: 5–7 (LOBA Chemie, Empirical formula: (C2H4O)x).
  PVA-based polymers offer outstanding chemical, mechanical, and biological properties, and no negative side effects are anticipated following their decomposition. PVA is white in color and exhibits abrasion resistance, high strength, corrosion resistance, and high hygroscopicity29.
3. Hyaluronic-acid MW-MD 3.5%: it is a biocompatible biomaterial that exhibits high biodegradability and extreme water solubility. Its molar mass is: 3–7X106 g/mol (Thunder company, Egypt, Empirical formula: (C14H21NO11) n).
  Hyaluronic acid is a naturally occurring biopolymer that serves a variety of purposes in the body, including cellular functions and wound healing. It has been a crucial part of biomedical research because of its adaptability in many applications in a variety of sectors, including tissue engineering 30. It acts in a similar manner to collagen fibers helping to increase the mechanical proprieties of medical sutures33.
4. Glycerin: It is a simple polyol hygroscopic compound with a density of 1.25 g/mL, molecular weight/Molar Mass: 92.09 g/mol (Pharmalog company, Egypt, Empirical formula:
  Although glycerin is typically thought of as an osmotic laxative, it can also have lubricating and softening properties. Additionally, scientific evidences prove that glycerin can inhibit bacterial growth 34.
  C3H8O3).
5. Sodium alginate: it is a constituent of the cellular wall in marine brown algae. It is comprised of approximately 30 to 60% alginic acid. The transformation of alginic acid into sodium alginate permits its dissolution in water, thereby facilitating its extraction.
  Additionally, scientific evidence proves that glycerin can inhibit bacterial growth. It is Empirical formula: (NaC6H7O6) is a linear polysaccharide derivative of alginic acid which comprised of 1,4-β-d-mannuronic (M) and α-l-guluronic (G) acids.
6. poly lactic acid (PLA): it is a biocompatible biomaterial that exhibits high biodegradability (Pharmalog company, Egypt, Empirical formula (C3H4O2) n).
  Its low melting point, high strength, low thermal expansion, good layer adhesion, and high heat resistance when annealed make it an ideal material for this purpose. Without annealing, however, PLA has the lowest heat resistance of the common 3D printing plastics.

All these additives are widely used for biomedical applications^6^, and are FDA approved polymers with good biocompatibility, biodegradability, and mechanical properties. Degradable materials such as PVA, Hyaluronic-acid, Glycerin, and water are non-toxic and can be excreted without harmful accumulation in tissues and organs.

### Extruder design

This section describes the design and structure of a small custom-made plastic extruder machine that will be used in production of filament sutures (FS) from the previously explained composites. The aim of the extruder machine is to convert the polymer extracted from human serum albumin to FS using the extrusion processing technique. During the process, the machine heats the polymer and extrudes a filament with a specific thickness. The extrusion machine has a construction that is similar to that of a commercial machine, but we designed and implemented it in our lab for cost effectiveness reasons.

**Figure 1:**
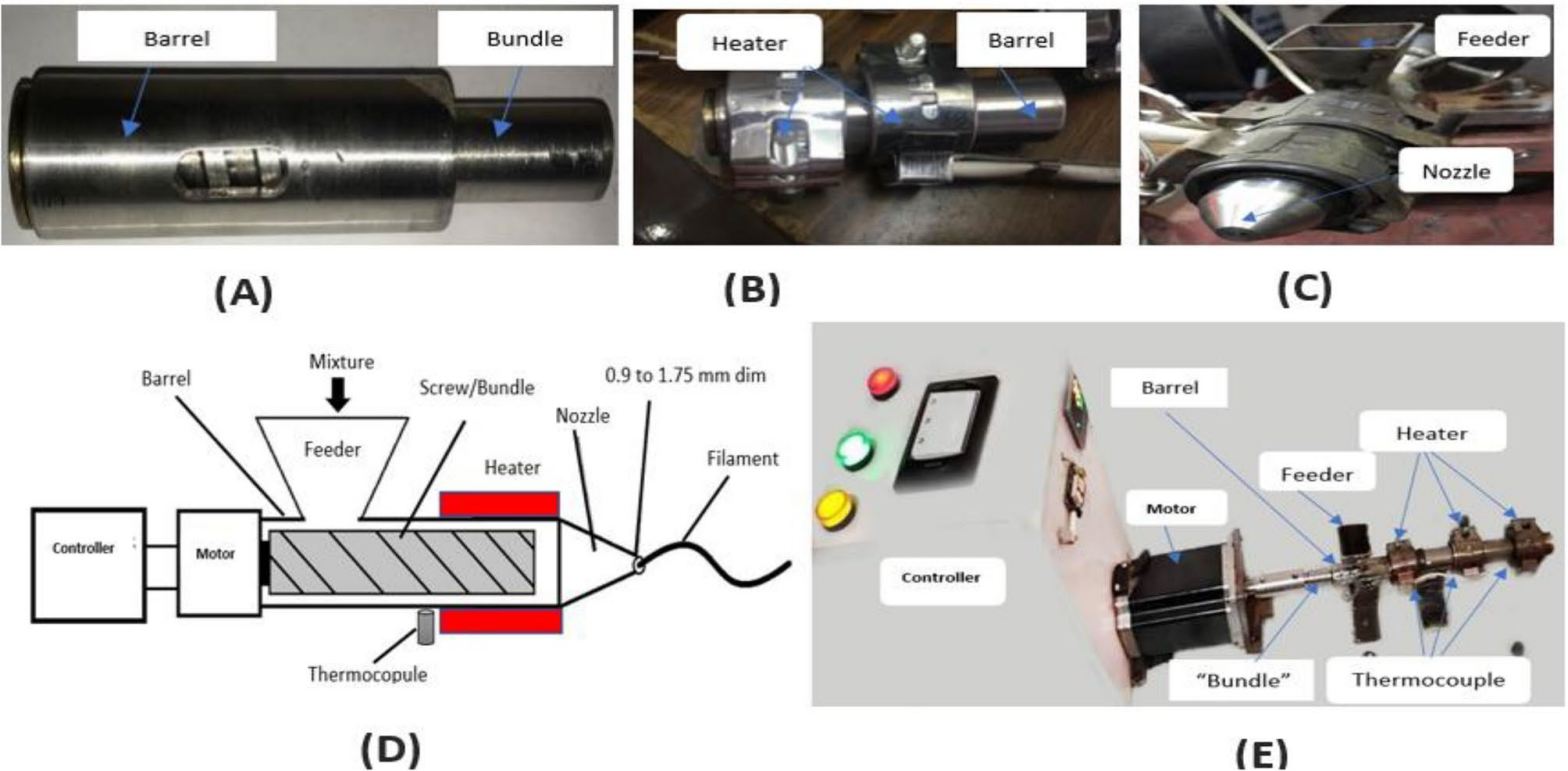
The developed custom-made extruder. (**A**) stainless steel bundle and barrel, (**B**) Bundle and barrel enclosed by heaters, (**C**) The inlet Feeder and nozzle (**D**) The assembly design, (**E**) Final extrusion device finishing.

The custom extruder main components were made of a barrel and a bundle, as shown in Fig. 1A, which are enclosed by a heater coil that is controlled by a temperature controller; Fig. 1B. To avoid reaction with the mixture materials, the barrel and bundle were made of 304 stainless steels. A high torque stepper motor provided mechanical power to rotate the stainless-steel screw 40N.cm and 200 steps/revolution (1.80/step) (Nema 34 closed Loop Stepper Motor 8.5Nm/1204 oz.in Encoder 1000CPR, Jiangning Nanjing City, China). Additionally, a closed loop stepper motor driver (CS-D1008, 20–100 VDC, Max 8A Output Current, Lead shine Technology Co., Ltd- China) was added to the system design to control the motor’s supply voltage; as shown in Fig. 1E. Motor directions and speeds were controlled with a custom lab design speed controller and an Arduino board. This design provided the necessary torque to generate enough pressure to force the mixture extrusion through the small output nozzle.

Figure 1D illustrates the main components of the developed extruder as a diagram. Figure 1E shows the final implemented design with an added conically shaped extruder’s end hole with nozzles of varying diameters. The material inlet opening was made wide enough (30 mm * 30 mm) to avoid wasting the mixture. The interspaces of the bundle’s teeth were kept small (12 mm) to increase the grinding effect. According to the temperature condition inside the barrel, the machine could melt the mixture as it flows through the nozzle opening, forming the albumin-based filaments.

### Composite preparation

This subsection describes four experiments with different concentrations. Those experiments aimed to find the best composite materials with acceptable temperature conditions to produce a biocompatible and high-quality suture. The four mixtures had fixed masses of the base materials: 5 g protein and 3 g water, while the other additive materials concentrations were changed in the experiments, as Table 1 shows. Firstly, our material experiments were aimed at obtaining the optimal temperature of the extrusion process while avoiding burning the composites. Previously, reported that the transition temperature of native Protein from SA is between 60 and 85 °C^23,24,33–35^. Therefore, the temperature for the extrusion process for the whole composites was maintained between 50 and 100 °C in order to achieve an acceptable concentration/viscosity without burning under vision inspection test. As shown in Table 1, from experiments, the best temperatures were achieved around 60–85 °C under vision test. From the other side, the Experimentation with the last mixture helped us obtain the optimal concentration ratios of raw materials to optimize for the mixture properties. So, the mixture 1.4 was exposed to many different trail with different heating temperatures.^25,26^ We assessed the experiments as following:

**Table 1:** Different concentrations are processed at different temperatures of four mixtures with the base materials protein (5 g) and water (3 g), while changing the additive materials.

Concentration no	Gelatin	Glycerin	Hyaluronic acid	Temp	Grinding condition	Mixture’s gradient
concentration 1	2 g	3 g	0	85°C	Electronic mill	Same gradient of mixture
concentration 2	2 g	3 g	0	60°C		
concentration 3	2 g	3 g	0	75°C		
concentration 4	3 g	2 g	2 g	60°C	Electronic mill	

The mixture’s gradient was constant concentration. While using manual blending for the mixtures, it causes the produced sutures were not be completely homogenous as it makes the thread appear granular. The protein was grounded by an electronic blender (Tornado electrical blender 500 W, MX5200/2) for 30 s, then the whole mixture was grounded for another 35 s while changing the temperature. concentration 1 was extruded at a temperature of 85 °C, concentration 2 was extruded at 60 °C, and concentration 3 was extruded at 75 °C.

After many trial and error attempts to reach an acceptable mixture concentration, mixtures of concentration 4 in Table 1 were found to exhibit a better tensile strength and elasticity than the other mixtures under manual inspection. The protein was grounded by blender machine for 35 s, the whole mixture was grounded for another 55 s, then the mixture was extruded at temperature 60 °C. The main goal of the previous experiments was to explore and determine the best temperature which maintains the best mixing concentrations and avoid burning to mixture. From point of burning condition, the output from the extruder of previous samples was changed according to the temperature conditions. The best accepted temperature for the mixture that avoids the burning was found to be around 60 °C that provides and acceptable filament’s flexibility, while higher temperatures caused burning of the mixture components.

### Tensile strength, SEM and TGA tests

Three testing methods were used to determine the mechanical and thermal properties of the produced sutures. First, manual tensile testing—by hand test- was applied on the samples before applying tensile testing machine. Scanning electron microscopy (SEM) imaging was performed on the samples to investigate their microscopic structure. Third, Thermogravimetric Analysis (TGA) testing was applied to assess the materials’ thermal stability and their fraction of volatile components as a function of temperature and time.

Although the tensile strength of the sutures produced from the Sample Type #1, shown in Table 2, was acceptable, as tensile tests show, their diameter was 1.75 mm which was relatively thick to be used in in vitro and in vivo experiments. Many sutures could be produced that achieved the desired diameter of lower than 0.5 mm, but the tensile strength might not be technically acceptable. To fulfill the suture’s requirements of being small in diameter with high tensile strength, the end nozzle’s opening of the extruder was modified to be 0.9 mm producing Sample Type #2, while changing the mixture components as shown in Table 4 to achieve the desired tensile strength shown in Table 5. All produced sutures were tested by tension testing machine, SEM, and TGA as will be described in the following section.

**Table 2:** sample type 1 different material compositions extruded using a 1.75 mm nozzle at 60 °C. The sample types were made with the base materials protein (5 g) and water (3 g), while changing the additive materials.

Sample no	Composite Concentrations			
	Gelatin	Glycerin	PVA	Hyaluronic acid
Sample 1.1	2 g	3 g	0	0
Sample 1.2	3 g	2 g	0	0
Sample 1.3	3 g	2 g	2 g	0
Sample 1.4	3 g	2 g	2 g	0
Sample 1.5	3 g	2 g	0	2 g

### Methodology of pharmaceutical tests

The cytotoxic effects of pHSA (created as a construct) were examined on various cell types: oral epithelial cells (OEC), human skin fibroblast cells (HSF), and human osteosarcoma cells (MG-63). These cells were sourced from Nawah Scientific Inc. in Mokatam, Cairo, Egypt, and were cultivated in DMEM medium enriched with 100 mg/mL streptomycin, 100 units/mL penicillin, and 10% fetal bovine serum that had been inactivated by heat. Cultivation conditions included a humidified atmosphere containing 5% CO2 at a temperature of 37 °C. The pHSA construct was administered to the cells over several time periods: 48 h (2 days), 72 h (3 days), and 168 h (7 days), with its cytotoxicity assessed via the sulforhodamine B (SRB) and WST-1 assays.

To evaluate cell survival, the SRB assay was applied over a 3-day period. A 100 μL cell suspension, with a density of 5 × 10^3 cells, was seeded into 96-well plates and incubated for 24 h in complete medium. Then, the cells were exposed to 100 μL of medium containing varying concentrations of the pHSA construct, from 10 to 100 μg/mL. After three days, the medium was replaced with 150 μL of 10% trichloroacetic acid (TCA) and chilled at 4 °C for one hour to fix the cells. The TCA was then discarded, and the cells were rinsed five times with distilled water. Afterwards, 70 μL of SRB solution (0.4% w/v) was added, and the mixture was incubated in the dark at room temperature for 10 min. After washing three times with 1% acetic acid and drying overnight, 150 μL of TRIS (10 mM) solution was used to dissolve the bound dye, and absorbance was measured at 540 nm using a BMG LABTECH®- FLUOstar Omega microplate reader (Ortenberg, Germany). The 7-day cell viability was similarly assessed using the SRB assay. Here, 1000 μL of cell suspension, containing 5 × 10^3^ cells, was incubated in 6-well plates with complete medium for 24 h, followed by treatment with 500 μL of medium containing the pHSA construct at concentrations ranging from 10 to 100 μg/mL. After 7 days, the cells were fixed with 1500 μL of 10% TCA, chilled at 4 °C for an hour, washed five times with distilled water, and then treated with 1000 μL of SRB solution. The plates were incubated, washed, dried, and the dye was dissolved as previously described, with absorbance read at 540 nm. Cell viability was also determined using the WST-1 assay for a 2-day period. For this assay, 1000 μL of cell suspension, containing 3 × 10^3^ cells, was placed in 6-well plates and incubated for 24 h. The cells were then exposed to 500 μL of medium with the pHSA construct at concentrations of 10 μg/mL and 100 μg/mL. After 48 h, 150 μL of WST-1 reagent was added, and absorbance was measured at 450 nm after 1 h using a BMG LABTECH®- FLUOstar Omega microplate reader, (Ortenberg, Germany). Cell viability was also assessed using the WST-1 assay for a duration of 2 days. This assessment was conducted using the Abcam® kit (ab155902 WST-1 Cell Proliferation Reagent)^36^. Aliquots comprising 1000 μL of cell suspension, which contained 3 × 103 cells, were introduced into 6-well plates and incubated in complete media for a duration of 24 h. Subsequently, the cells were subjected to treatment with an additional aliquot of 500 μL of media, which contained the PBSA construct at varying concentrations (10 ug/ml, 100 ug/ml). Following a 48-h exposure to the drug, the cells were treated with 150 μL of WST-1 reagent, and the resulting absorbance was measured after 1 h at a wavelength of 450 nm, employing a BMG LABTECH®- FLUOstar Omega microplate reader located in Allmendgrün, Ortenberg.

## Results and discussion

Suturing materials were prepared in a sterile environment, with a continuous ribbon, and were employed in the laboratory for production of the FS materials. In this section, we introduce the results of two experiments in an effort to optimize the suture’s mixtures.

### Samples type 1 testing results

**Figure 2:**
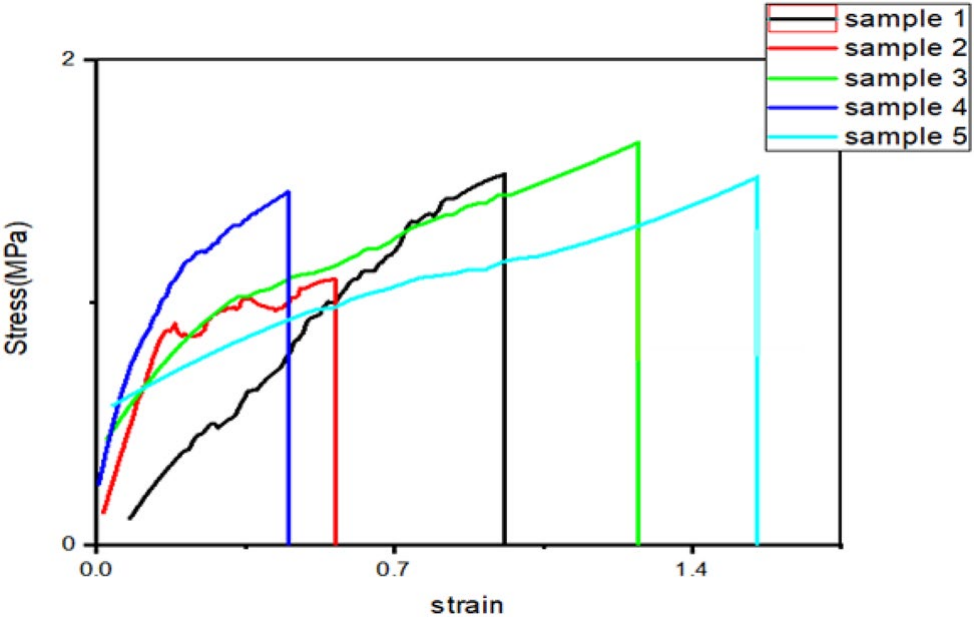
Stress–Strain curves for suture samples of Type 1.

**Figure 3:**
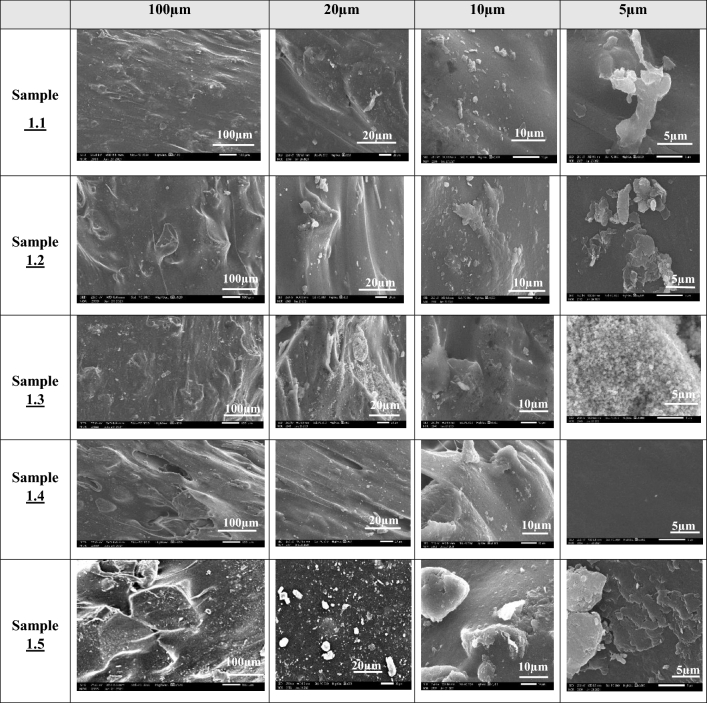
SEM images of suture samples of Type 1 produced using a 1.75 mm nozzle. White bars represent the corresponding scale for each imaging magnification.

**Figure 4:**
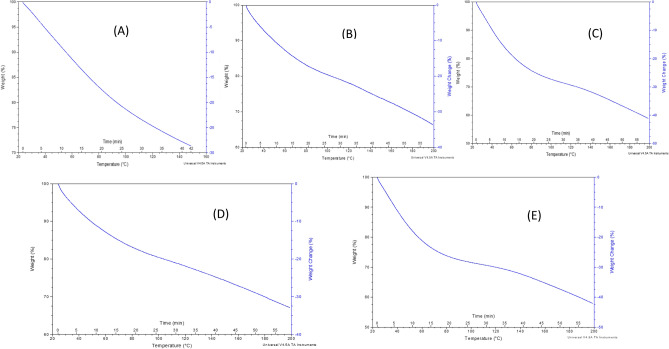
Type 1 samples thermal stability analysis: TGA curves for sutures produced using the 1.75 mm nozzle: (**A**) Sample 1.1. (**B**) Sample 1.2. (**C**) Sample 1.3. (**D**) Sample 1.4. (**E**) Sample 1.5

Five samples were tested via tension testing, SEM, and TGA techniques, as shown in Table 3 and Figs. 2, 3 and 4.

**Table 3:** Samples type 1 mechanical proprieties of sutures produced from nozzle 1.75 mm.

	Sample 1.1	Sample 1.2	Sample 1.3	Sample 1.4	Sample 1.5
Elongation %	96.603	49.559	126.85	44.880	146.64
Maximum Load (N)	2.2312	2.1797	3.5278	3.4631	2.8892
Tensile Strength at max load (MPa)	1.8993	1.3764	1.9188	1.6334	1.7765

#### Tensile strength results of type 1 samples

Tensile experiments were performed on the FS samples using the tensile device (LLOYD –LRSK PLUS SERIES materials testing machine, Lloyd Instruments Ltd, UK). It is noticeable that Sample 1.3, which has PVA, showed the highest ultimate tensile stress and young’s modulus than other samples, while sample 1.5, which have hyaluronic acid, achieved the highest toughness. Additionally, only samples 1.3 and 1.5 exhibited an elongation ratio of more than 100%, with 1.5 having stretchability of about 150%. Table 3 summarizes the mechanical proprieties of the synthesized filament sutures from nozzle 1.75 mm.

#### SEM and TGA results of samples type 1

SEM characterization was performed using a scanning electron microscope (JSM-IT200, JEOL InTouch Scope™ series, Tokyo, JAPAN). Figure 3 provides the SEM images of type 1 samples (thickness of 1.75 mm) at different scales. All mixtures of these samples contained Glycerin and gelatin, in which PHSA are mixed and integrated together to form a gel. It is noticed in the SEM images of Sample 1.4 that the granular structure of the suture is more homogeneous with less scattered granules and well oriented surface, compared to the other sutures, which could indicate that the material was mixed in a better way than the other Samples of type 1. From Fig. 3, it is shown that the granules in Samples 1.1, 1.2, 1.3 and 1.5 are not completely homogeneous. Thermogravimetric analyzer TGA (TGA THERMOSTEP device, ELTRA GmbH part of verder scientific group, Netherlands), in Fig. 4 show the thermal stability of the produced FS. It is noticed that these types of samples did not lose more than 5% of their original weight when heated for 5 min at the working temperature of about 370. It is noticed also that the samples 1.2 and 1.5 are the best samples for weight loss as they loss less than 35% weight change at temperature 200 °C. Sample 1.1 losses 27% weight change at temperature 150 °C. Sample 1.3 losses more than 40% weight change at temperature 200 °C. Sample 1.4 losses more than 40% weight change at temperature 200 °C.

### Samples type 2 testing results

Our research team tried some other additions according to mechanical results of suture type 1, shown in Table 3, including gelatin, Sodium alginate and poly lactic acid (PLA) polyvinyl alcohol, and hyaluronic acid, as shown in Table 4, while extruding these samples with 0.9 mm nozzles to produce samples with smaller diameters that fit more the purpose of suturing. The four samples were tested via the tension test, SEM, and TGA techniques.

**Table 4:** Sample Type 2: Different compositions extruded using a 0.9 mm nozzle at 60 °C.Base materials used were protein (5 g), water (3 g), Sodium alginate (0.22 g) and poly lactic acid (PLA) (0.73 g), while the additive materials were changed.

Sample No	Composite Concentrations			
	Gelatin	Glycerin	PVA	Hyaluronic acid
Sample 2.1	2 g	3 g	0	0
Sample 2.2	3 g	2 g	0	2 g
Sample 2.3	3 g	2 g	2 g	0
Sample 2.4	3 g	2 g	2 g	2 g

#### Tensile strength results of samples type 2

Tensile experiments were performed on samples of the second type with 0.9 diameter using the tensile device (Instron single column, system ID:3345L8714, USA). It was noticeable that during tensile strength testing of the samples, we noticed that adding (PVA + Hyaluronic Acid) in the Sample 2.4, increase the tensile strength and elongation % in agreement with the rest of the sutures testing and with the examination of the sutures over time, we noticed that the tensile strength increased as the sample was subjected to further cooling. As shown in Table 5, the tensile stress and Elongation percentage of Sample 2.4 was 9.616 MPa and 38, respectively and higher than the other tensile stress and Elongation percentage of Samples (1, 2 and 3). These observations confirm that adding PVA and hyaluronic acid (based collagen) to the PHSA and gelatin solvent by water and glycerin is highly recommended to achieve the required tensile strength.

**Table 5:** Sample type 2: mechanical proprieties of sutures produced from nozzle 0.9 mm.

	Sample 2.1	Sample 2.2	Sample 2.3	Sample 2.4
Elongation %	14.5	11.5	36	38
Max load (N)	5.044	21.223	17.001	16.993
Tensile stress at max load (MPa)	5.3086	7.727	6.393	9.616

#### SEM and TGA results of samples type 2

**Figure 5:**
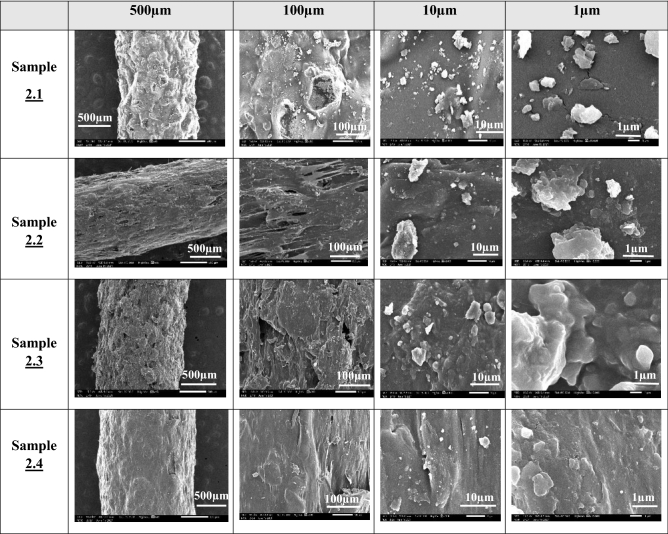
SEM images of suture samples of Type 1 produced using 0.9 mm nozzle. White bars represent the corresponding scale for each imaging magnification.

**Figure 6:**
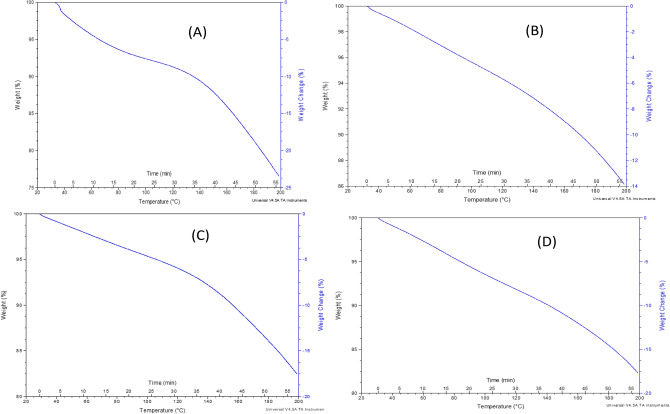
Type 2 samples thermal stability analysis: TGA curves for sutures produced using the 0.9 mm nozzle: (**A**) Sample 2.1. (**B**) Sample 2.2. (**C**) Sample 2.3. (**D**) Sample 2.4.

SEM characterization was performed by using a JSM-IT200 scanning electron microscope. Figure 5 shows the SEM image of Samples type 2 (with a thickness of 0.9 mm) at temp 60 °C carried using gelatin, in which PHSA are mixed and integrated together to form a gel. From Table 3, it is noticed that the effects of mechanical proprieties of PVA and Hyaluronic acid. SO, in these mixtures, PVA was used in Samples 2.3 and 2.4, hyaluronic acid was used in Samples 2.2 and 2.4, while Sample 2.1 did not have PVA and hyaluronic acid as mixing components. Figure 5 shows that the morphology of the mixture that has hyaluronic acid and PVA in Sample 2.4 exhibits a smoother surface profile. The granularity in Sample 2.4 showed that the material was mixed well and better than the other sutures, which is clear at scale 500 µm. TGA tests have shown the thermal stability of the FS, particularly Sample 2.3. The first weight loss occurs due to water molecules evaporation, whereas the second weight loss is due to the degradation of collagen. It is noticed from TGA Fig. 6 that the samples type 2 are better than samples type 1 regarding weight loss at higher temperature. Sample 2.1 losses more than 23% weight change at temperature 200 °C. Sample 2.2 losses more than 13% weight change near a temperature 200 °C. Sample 2.3 losses more than 16% weight change near a temperature 200 °C. Sample 2.4 losses more than 16% weight change near a temperature 200 °C.

**Figure 7:**
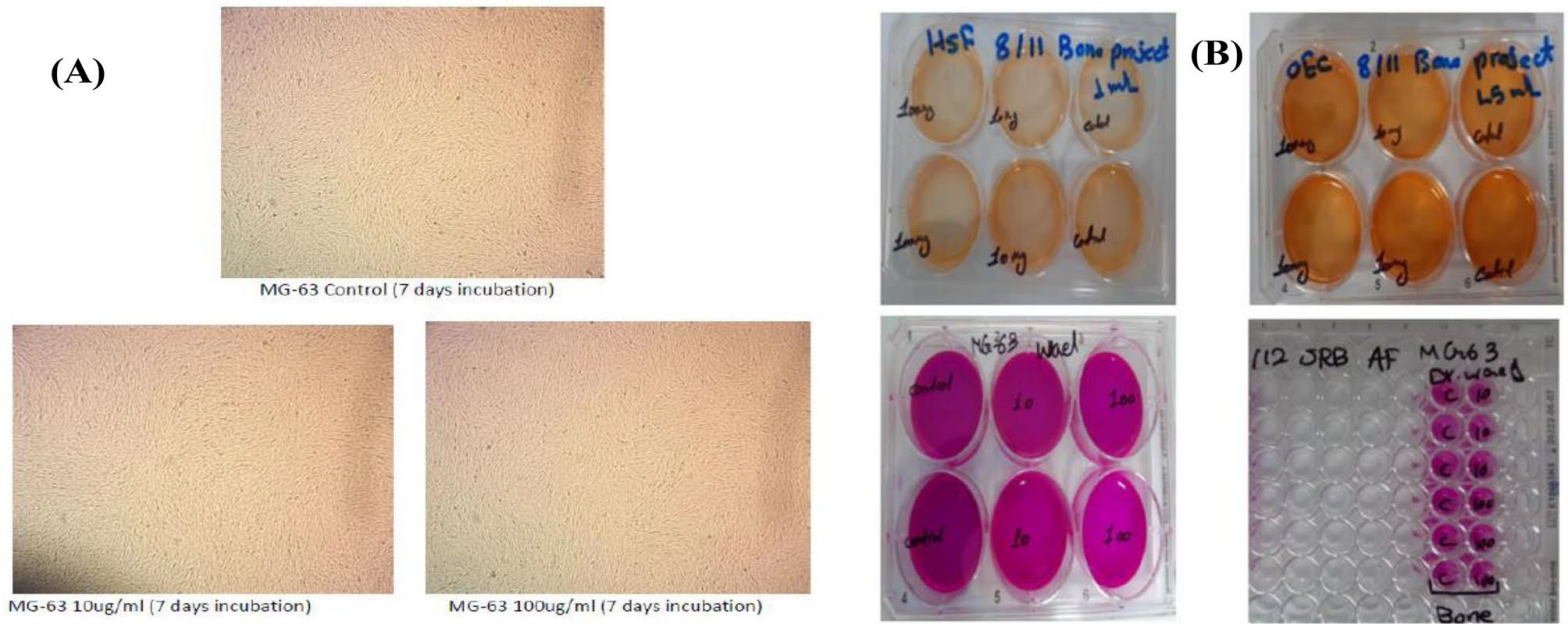
Cytotoxicity tests: (**A**) MG_63 samples during incubation. (**B**) OEC, HSF and MG-63 samples. Tested cells showed regular growth pattern and normal proliferation rate during the incubation periods.

Table 6 shows the percentage of viable cells after the incubation period in comparison with the control. Oral epithelial cells and human skin fibroblast cells treated with bone graft sample (PHSA with porous structure of 0.3 g catalyst) at high and low concentrations, have showed normal rate of proliferation when compared to the control cells. Moreover, long incubation periods were conducted on MG-63 bone cells to ensure the safety of the sample. After three days and seven days incubation, there was no significant difference in the percentage of viable cells between the treated and the control cells. In comparison to the control, it could be concluded that the bone graft sample has no cytotoxic effects on cells; and OEC, HSF and MG-63 had regular growth pattern and normal proliferation rate during the incubation periods as shown in Fig. 7A,B.

**Table 6:** The percentage of viable cells after the incubation.

Bone concentration (ug/ml)	SRB_ MG-63 (3 days)	SRB_ MG-63 (7 days)	WST1_ OEC (2 days)	WST1_ HSF (2 days)
10	94.33%	90.60%	99.78%	99.47%
100	94.19%	89.06%	96.51%	93.09%

## Discussion

The primary goal of our research team is to validate the feasibility of converting protein derived from human serum albumin into filaments that possess superior mechanical properties and can be effectively utilized in the field of medicine. It is important to note that our research team is recognized as the pioneering group that has endeavored to utilize protein sourced from human serum albumin. Therefore, there is no specific reference available to determine the concentration proportions of the mixture used in the process of fabricating filament sutures through the extrusion machine developed by our research team. As a result, we had to rely on the method of experimental investigation, involving various concentrations, in order to achieve the optimal outcomes.

Those processing steps were first optimized by a trial-and-error routine, since the product is new and there was no similar product in the market to be taken as a guide. After many trials we could.

adapt a simple and straight forward strategy for the processing methodology. Then, the processed suture FS was used for evaluating the different mechanical and thermal properties.

*Part #1* The primary objective of investigating the concentration of Suture Type 1 was to determine the optimal temperature for the mixture using accepted mechanical properties. This resulted in the production of filament sutures with a diameter of 1.75 mm. However, attempts to reduce the diameter of the filament sutures, while maintaining the same concentration as in Suture Type 1, led to a decrease in the mechanical properties, particularly in the tension process. Therefore, it is necessary to change the concentration of the mixture.

*Part #2* In order to improve the mechanical properties of filament sutures with a diameter smaller than 1.75 mm, the concentration of Suture Type 1 was adjusted by incorporating additional biodegradable materials that possess significant chemical and physical properties. This resulted in the creation of Suture Type 2 with a diameter of 0.9 mm. However, sutures currently available in the industry have relatively small diameters, but certain types have limited mechanical characteristics. Therefore, our team is currently conducting ongoing research to modify the properties of sutures while simultaneously reducing the diameter to meet commercial standards.

SEM technique was performed on all output sutures beginning by samples 1 producing poorly distributed particles which encouraged us to modify its concentration to form sutures sample 2 trying to modify the mechanical proprieties conditioned by decreasing the diameter size from 1.75 mm to 0.9 mm. However, the comparable suture in the industry were characteristic by small scale diameters, but some types have limited mechanical characteristics,^37–40^. So, our team is still working, as a future work, on modifying the characteristics of Sutures while minimizing the diameter to reach the commercial standards.

While the results show the low tensile strength of the produced sutures-based albumin compared to commercial sutures, this study motivates us to work more on the development of albumin-based sutures. An important advantage of developing sutures from serum albumin is adding protein to the body during the biodegradation process. Serum albumin is consider one of the most important biochemical indicators of malnutrition ^41,42^ .The distinctive properties of FS are that it shows tensile strength of tested sutures varies from 1.3 to 9.616 (Mpa) and 11.5 to 146.64 (%) as elongation at break. Rethinam et al.^43^ investigated a unique multifilament suture that was prepared from animal skin fiber that reported a tensile strength of 85.25 ± 0.09 (Mpa), 81.9 ± 0.46(%) as elongation at break. Rethinam et al. ^5^ developed an absorbable suture that it exhibits tensile strength as high as 43.16 ± 1.03 MPa which have a potential wound healing as well as external surgical applications. Rethinam et al.^44^ reported a biocompatible polymeric bio-scaffold that can significantly accelerate the process of wound healing which its mechanical characteristics show tensile strength of 20.88 ± 0.05 (Mpa), 17.74 ± 0.05 (%) as Elongation at break.

Zhang et al.^45^ produced fibers based on natural sources including chicken egg, quail egg, goose.

egg, milk, bovine serum albumin and collagen that can be used in suturing applications in rat and minipig models depend on the microfluidic device. The breaking strengths of the quail and goose egg white fibers were about 44.50 and 27.83 MPa, respectively. after crosslinking with glutaraldehyde, those values slightly increased to 47.84 and 29.25 MPa. The fibers-based milk showed a breaking strength of about 48 MPa which increased to 75 MPa after crosslinking of glutaraldehyde. Remarkably, the authors reported that following the glutaraldehyde crosslinking treatment, a significant increase in the mechanical performance of the BSA fibers was observed. The breaking strength of the BSA fiber increased from 55.24 to 99.28 MPa. Based on previous works, our research team aims to prove that the protein from human serum albumin can convert to sutures with good mechanical proprieties that can be used in medical branches.

This study presents two important topics. The first is the design of custom extruder machine and the second is developing new filament suture based on serum albumin to aid in the FS production and maintain the extrusion process. It is well known that a medical suture should not only be very strong with high mechanical properties, but also be able to simply disintegrate in body fluids and lose strength at the same rate as the tissue is being repaired. It would neither cause nor promote complications.^46^.

Biodegradable sutures based on albumin composites play a crucial role in the field of green biomaterials due to their sustainability and biocompatibility. The following paragraph discusses the key points related to green biomaterials^47^.

*Sustainability* Albumin composites for sutures are sourced from natural origins like bovine or human serum albumin, rendering them inherently more sustainable compared to synthetic alternatives derived from petrochemicals. This is in line with the principles of green chemistry and sustainable development ^26^.

*Biodegradability* One of the key advantages of biodegradable sutures is that they undergo degradation within the body over time, thereby eliminating the need for surgical removal. Albumin-based sutures experience enzymatic degradation by proteolytic enzymes found in the body, resulting in minimal tissue reaction, and facilitating wound healing^25,48^.

*Biocompatibility* Albumin is a protein naturally found in the human body, making albumin-based sutures highly biocompatible. This reduces the risk of adverse reactions, inflammation, and infections at the wound site. Additionally, albumin has been shown to promote cell adhesion and proliferation, further aiding in tissue regeneration^25,44^.

*Mechanical Properties* Albumin composites can be designed to have adjustable mechanical properties, ensuring they meet the requirements of various surgical procedures and tissue types^24,48^.

*Drug Delivery* Albumin-based sutures can also function as carriers for drug delivery, providing localized and sustained release of therapeutic agents or growth factors directly to the wound site. This can enhance the healing process and reduce the necessity for additional medication^49,50^.

*Reduced Environmental Impact* In comparison to non-biodegradable sutures, which have the potential to persist in the environment and contribute to pollution, biodegradable albumin-based sutures have a minimal impact on the environment. They undergo decomposition into natural byproducts, thus reducing the accumulation of medical waste^51^.

These sutures provide a combination of sustainability, biocompatibility, and mechanical properties that are essential for effective wound closure and tissue regeneration, while also reducing the environmental impact associated with medical waste.

Abdelmoez et al.^25,26,48^ reported that the transition temperature of native BSA was 64 °C. The authors found that the transition temperatures for PBSA samples were 66, 77, 78, and 85.5 °C, respectively. So, the temperature for the extrusion process was maintained between 60 and 85 °C in order to achieve the optimal temperature avoid burning the mixture based on trial-and-error method. The accepted temperature for the mixture was found to be 60 °C ± 5 that provides and acceptable filament’s flexibility, while higher temperatures caused burning of the mixture components. The denaturation process of protein is governed by two factors, namely the temperature and the duration of heat exposure. According to the research conducted by Borzova, Vera et al.^52^, (BSA) protein denaturation can occur for a duration exceeding 12 min at a temperature of 65 °C. However, in our investigation, the mixture is subjected to a temperature of 65 °C ± 5 for a duration of less than one minute, thereby reducing the likelihood of protein denaturation to a level lower than anticipated. The data from Table 6 and Fig. 7A,B suggest that the bone graft sample exhibits no cytotoxic effects when compared to the control group. This outcome implies that, within the confines of the conducted experiment or study, the bone graft material poses no risk to cellular health, maintaining normal growth patterns in OEC, HSF, and MG-63 cells. The previous results encourage us to continue our research efforts to modify the FS regarding its strength and size to make it a more practical product as surgical biodegradable sutures.

## Conclusion

To the best of our knowledge, this is the first time that sutures can be constructed based on proteins from human serum albumin derived under subcritical water technology. The novel filament sutures were extensively constructed by the hot-melt extrusion process to successfully develop a biopolymer composite. The strategy used in this study has a lot of promise since the melt extrusion technique is relatively versatile, inexpensive, and simple to scale up. FS created potential biomaterials that can be used for multiple biomedical applications, including suturing materials and wound dressing. The developed suture was produced from human serum albumen and can be used for wound closure. The physicochemical and mechanical tests were conducted and showed promising results.

Smaller sutures diameters will be required in future works. In vivo and biodegradation tests are also required to validate suture degradation with an appropriate tissue regeneration. One of the most interesting applications of the proposed suture is the ability to be used as a 3D printer filament to print custom scaffolds, nails, and plates with carefully design properties using finite element modelling.

## Data Availability

All data and materials are the property of the authors. Corresponding authors may provide raw data upon reasonable request.
